# Effect of Particle Specific Surface Area on the Rheology of Non-Brownian Silica Suspensions

**DOI:** 10.3390/ma13204628

**Published:** 2020-10-16

**Authors:** Anastasia Papadopoulou, Jurriaan J. J. Gillissen, Manish K. Tiwari, Stavroula Balabani

**Affiliations:** 1 FluME, Department of Mechanical Engineering, University College London, Torrington Place, London WC1E 7JE, UK; anastasia.papadopoulou.16@ucl.ac.uk; 2 Nanoengineered Systems Laboratory, Department of Mechanical Engineering, University College London, Torrington Place, London WC1E 7JE, UK; m.tiwari@ucl.ac.uk; 3 Department of Mathematics, University College London, Gower Street, London WC1E 6BT, UK; jurriaangillissen@gmail.com; 4 Welcome/EPSRC Centre for Interventional and Surgical Sciences (WEISS), University College London, London W1W 7TS, UK

**Keywords:** commercial silicas, surface roughness, particle porosity, glycerol, shear rheology, specific surface area

## Abstract

Industrial formulations very often involve particles with a broad range of surface characteristics and size distributions. Particle surface asperities (roughness) and porosity increase particle specific surface area and significantly alter suspension rheology, which can be detrimental to the quality of the end product. We examine the rheological properties of two types of non-Brownian, commercial precipitated silicas, with varying specific surface area, namely PS52 and PS226, suspended in a non-aqueous solvent, glycerol, and compare them against those of glass sphere suspensions (GS2) with a similar size distribution. A non-monotonic effect of the specific surface area (S) on suspension rheology is observed, whereby PS52 particles in glycerol are found to exhibit strong shear thinning response, whereas such response is suppressed for glass sphere and PS226 particle suspensions. This behaviour is attributed to the competing mechanisms of particle–particle and particle–solvent interactions. In particular, increasing the specific surface area beyond a certain value results in the repulsive interparticle hydration forces (solvation forces) induced by glycerol overcoming particle frictional contacts and suppressing shear thinning; this is evidenced by the response of the highest specific surface area particles PS226. The study demonstrates the potential of using particle specific surface area as a means to tune the rheology of non-Brownian silica particle suspensions.

## 1. Introduction

Silica particles with high degrees of surface roughness and porosity, as well as wide size distributions, are commonly used in industrial formulations and everyday consumer products. Such characteristics entail complex particle dynamics [1,2,3], which are poorly understood and can further complicate suspension rheology. Precipitated silicas are mainly produced through the reaction of an aqueous sodium silicate solution with mineral acids. The amorphous silicon dioxide particles comprise primary nanoparticles with diameter of ~10 nm, which are instantly fused together into stable irregularly shaped aggregates with increased surface roughness and porosity, leading to high particle specific surface areas (S). The aggregates can effectively serve as larger particles with a high degree of surface asperities (roughness) and porosity. 

The particle surface asperities (roughness), porosity, and specific surface area appear to be closely linked. In particular, the more irregularly shaped and porous the particles are, the higher their specific surface area is. Although the latter could potentially serve as a parameter expressing the combined effects of particle surface asperities and porosity, its role on suspension rheology is generally overlooked with only a few and rather contradictory relevant studies appearing in the literature. For example, Asija et al. [4] used fumed silicas suspended in polyethylene glycol and reported an increase in the shear thinning response and a decrease in the shear thickening with increasing the specific surface area of the particles from 90 to 200 m^2^/g. However, the weakening of the shear thickening was attributed to the smaller average primary particle size associated with the higher surface area, which resulted in milder hydrodynamic forces unable to overcome the repulsive forces and induce particle hydroclustering, and hence shear thickening [4]. Other studies employing fumed silicas with varying specific surface areas from 200 to 380 m^2^/g in organic and inorganic solvents did not report any significant effects of this parameter on suspension rheological properties [5,6,7]. However, fumed silicas are colloidal, and thus the Brownian effects are likely to govern their suspension rheology rather than the particle specific surface area.

On the other hand, the effect of particle surface roughness on the rheology of non-colloidal suspensions has been studied by several researchers [2,3,8,9]. Increasing particle surface roughness has been found to increase suspension viscosity and has led to pronounced non-Newtonian rheology at lower particle volume fractions as compared with smooth spheres due to increased frictional surface contacts between the particles. Moon et al. controlled the particle surface roughness through mechanical grinding [3]. Smooth and rough non-Brownian polymethylmethacrylate (PMMA, D=40 μm) and polystyrene (PS, D=40 & 80 μm) were suspended in silicone oil. Rough particles induced a 20% to 50% increase in suspension viscosity as compared with their smooth counterparts, due to an increase in the available areas for particle surface contacts [3]. The work of Moon et al. [3] was complemented by Tanner and Dai [2] who induced a higher roughness ratio to the particles, up to 5%. At such high degrees of surface roughness, the particles lost their sphericity and particle contacts were favoured through the extended surface asperities [2,10]. Hoyle et al. also produced glass spheres (d~30 μm) with controlled roughness through wet chemical etching using hydrochloric acid (HCl) and sodium hydroxide etching. A silicone oil matrix was employed as the suspending medium. The shear thinning rheology of such suspensions was related to a variable friction coefficient resulting from the frictional contacts between the particles at increasing shear rate [8]. The friction coefficient of the suspensions of smooth and rough particles were estimated using the bootstrap method introduced by Tanner et al. [11]. This method suggests a relation between the microscopic friction coefficient and the suspension viscosity, the particle pressure and the measured shear stress [11]. In our recent studies, we also showed that the shear thinning response of suspensions of rough silica particles in glycerol could stem from the aforementioned friction-driven mechanism. It was also observed that particle surface roughness increased suspension viscosity and even promoted particle agglomeration due to increased interparticle surface contacts [12,13]. 

Precipitated silicas comprise aggregates of primary nanoparticles, and hence exhibit a high degree of surface asperities and porosity. Blanc et al. investigated the rheology of sugar particles (d~80–100 μm) with irregular shapes (faceted) as compared with relatively smooth and monodisperse polystyrene spheres (d~80 μm,), and suspended both in a Newtonian silicone oil matrix [9]. The irregular shape of the faceted particles was found to increase the zero shear viscosities and the shear thinning nature of the suspensions as compared with the polystyrene spheres at the same particle volume fractions. The non-Newtonian rheology of the faceted particles was attributed to the higher contribution of the contact viscosity to the total suspension viscosity value at increasing particle volume fractions, since particle contacts were favoured in the case of irregularly shaped particles [9]. Particle porosity has also been observed to significantly increase suspension viscosity through an increase in the effective particle volume fraction as compared with the apparent one, as the pores absorb part of the solvent [14,15]. 

The inherently hydrophilic silicas include certain active groups on their surface, namely free silanol groups (Si-OH), which enable particle–particle and particle–solvent interactions through hydrogen bonding. The density of the surface silanol groups depends linearly on the specific surface area, S [16,17,18]. When suspended in polar solvents, a solvation layer around the silicas is likely to form through hydrogen bonding between the hydroxyl groups (-OH) of the solvent’s molecules and the silanol groups on the silica surface, acting as a lubricant and preventing direct particle contacts. The strength and thickness of the solvation layer depends on several factors including the viscosity of the solvent and its ability to effectively bind on the silica surface, as well as the density of the silanol groups on the silica surface [7,19,20]. Therefore, the suspension rheology in such systems results from the competition between the particle–particle and particle–solvent interactions [21]. A high particle-solvent affinity leads to stable solutions where the particles stay well dispersed in the suspending medium repelling each other through the solvation layer. In contrast, when particle–particle interactions dominate, particle agglomeration is likely to occur resulting in highly elastic gels [7,22]. Gao et al. were able to measure the thickness of this solvation layer for fumed silicas suspended in an aqueous solvent using the small angle neutron scattering (SANS) technique. The measured thickness was found to span a few nanometers with a maximum value of 3 nm at room temperature [23]. 

Amiri et al. studied the rheology of fumed silica suspensions in water/glycerol solutions. In this study, the addition of glycerol to the aqueous solvent led to an increase in the stability of the suspensions, despite a decrease in the z-potential [19]. This behaviour was related to the existence of a thicker layer around the particles formed by glycerol, due to an increase in the contact sites as glycerol has three hydroxyl groups on its molecule to bind onto the silica surface as compared with water which has one. Saint-Michel et al., however, observed pronounced shear thinning in suspensions of non-Brownian glass spheres in an aqueous Newtonian solvent at φ≥0.30, arising from the interparticle contacts enabled in water due to a thinner solvation layer [20]. 

The present work addresses the role of specific surface area on the rheology of non-Brownian silica particles, in an attempt to elucidate the competition between particle–particle and particle–solvent interactions and potentially combine the effects of particle surface asperities and porosity of non-idealized particles, commonly employed in industry, into a single parameter. A non-aqueous polar solvent, glycerol, is used as the suspending medium. Using steady state and oscillatory shear frequency sweeps, we demonstrate a non-monotonic influence of the particle specific surface area on silica suspension rheology; the latter is discussed in relation to its influence on particle–particle and particle–solvent interactions, as well as effective volume fraction. 

## 2. Materials and Methods 

### 2.1. Particle Suspensions

**Figure 1:**
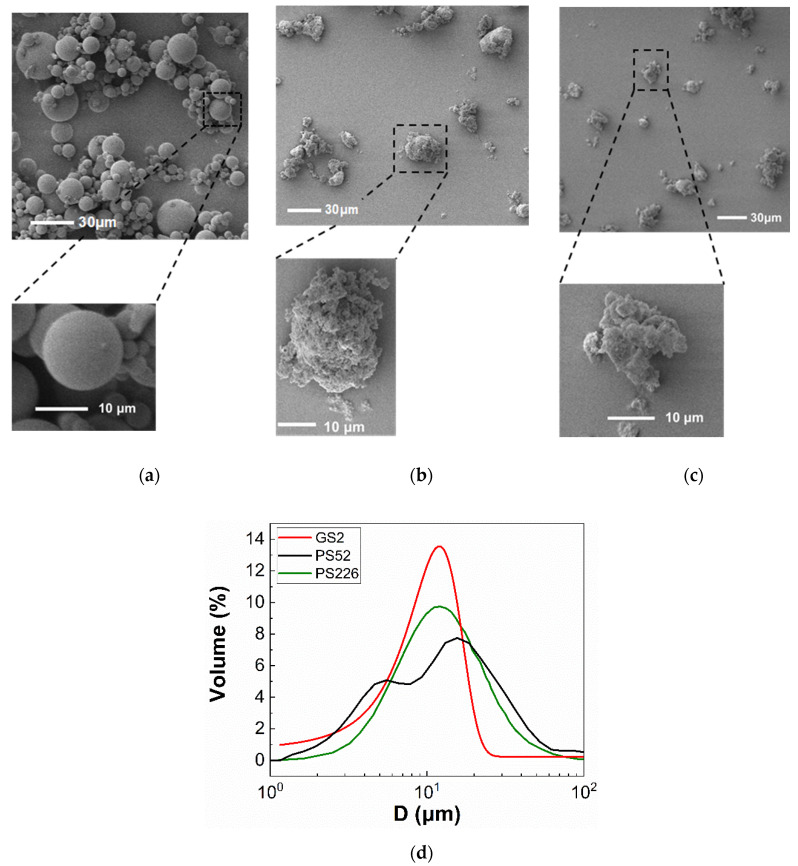
SEM micrographs of the three types of particles used in the present study. (**a**) Glass spheres (GS2); (**b**) Silica particles PS52; (**c**) Silica particles PS226. (**d**) Size distribution of the particles as derived from laser diffraction measurement.

Two types of commercially available silica particles, supplied in-kind by GlaxoSmithKline (GSK) and used as abrasives (D=17.5±15.2 μm) and fillers (D=12.5±4.0 μm) in oral healthcare formulations, and hollow glass spheres (D=11±5.5 μm) (Sigma-Aldrich) were suspended in glycerol (≥99.5%) (Sigma-Aldrich). The silica particles used in the present study comprise aggregates of primary particles with diameter ~10 nm, which are instantly clustered together during production (Figure 1b,c); the latter can partially explain their surface morphology. 

The particle properties were characterized using a suite of techniques, i.e., scanning electron microscopy (SEM) for surface morphology (Carl Zeiss Evo 25, Oxford Instruments, Abingdon, UK) and laser diffraction to determine the size distributions (Sync, Microtrac, Haan/Duesseldorf, Germany). Prior to SEM imaging, the particles were coated with a gold layer to increase sample conductivity. The specific surface area and porosity were estimated through the Brunauer–Emmet–Teller (BET) method using the 3 Flex instrument (Micromeritics). BET is a surface characterization technique based on the physical absorption of gas molecules on a solid surface in a monolayer form under the effect of pressure. Utilizing the data of the relative pressure in the system and the mass of the gas molecules being absorbed, the specific surface area (S) and the porosity (ε) of the particles can be estimated. The S and ε parameters highly depend on the way the aggregates of the commercial silicas are formed. The particle and glycerol properties are summarized in Table 1. Selected SEM micrographs and the corresponding size distributions of the three types of particles are shown in Figure 1. The silica particles are noted as PS52 and PS226, respectively, indicating that they are precipitated silicas with specific surface areas of $S\approx 52{\;m}^{2}/g$ and $S\approx 226{\;m}^{2}/g$, while the glass spheres are denoted as GS2, which stands for glass spheres with a specific surface area of $S\approx 2{\;m}^{2}/g$.

### 2.2. Sample Preparation 

A high shear mixer (Silverson, Model L5M, Chesham, UK) was used to disperse the required mass of solids for a certain particle volume fraction in glycerol. The samples were mixed at speeds of 3500 and 4000 rpm for 5 to 10 min depending on the particle type and volume fraction. A wide range of particle concentrations was used varying from 0 to 62% *v/v* for the glass spheres, resulting in particle volume fractions (φ) of 0 to 0.62. The maximum φ achieved for the commercial silicas was lower than that of the glass spheres; this was based on visual observations to ensure that the mixture was homogeneous, and no clumps of undispersed powder were present. This maximum dispersion volume fraction should not be related to the maximum packing fraction ($\varphi_{m}$) estimated through fittings to the data. The samples were placed in a vacuum chamber to remove the air bubbles entrapped during mixing and the rheological measurements were conducted right after the degassing stage to avoid particle sedimentation effects.

### 2.3. Shear Rheology

Steady state and oscillatory shear measurements were performed using a DHR-3 stress controlled rotational rheometer (TA Instruments, New Castle, DE, USA) equipped with a sandblasted (R_a_ = 1.8–2 μm) parallel plate geometry of diameter 40 mm. The gap between the parallel plates was kept at 650 ± 50 μm, to allow a sufficiently large number of particles to be accommodated within this space. The lower plate utilised a Peltier system to keep the temperature constant at 20 °C with an accuracy of ± 0.1 °C, while a solvent evaporation trap was also used to prevent the sample from evaporating. 

First, a pre-shearing step was conducted at a shear rate of 200 s^−1^ for 5 min to eliminate any prior shearing history in the material, followed by a 5 min resting period to allow restoration of properties [24,25,26]. Nondestructive oscillatory frequency sweeps were performed in the linear viscoelastic region (γ=0.1%) and at angular frequencies varying from 1 to 600 rad/sec. Flow sweeps under steady state followed at increasing shear rates ($\dot{\gamma }$) from 10^−3^ to 10^3^ s^−1^. The repeatability of the results was evaluated by conducting all measurements in triplicate. To correct for the non-uniform shear rate across the parallel plate geometry the Bowditch–Rabinowitch correction (Equation (1)) was applied to the data after acquisition [27] as follows:

(1) $$\tau_{true}=\frac{M}{2\pi R^{3}}\left(3+\frac{dlnM}{dln{\dot{\gamma }}_{R}}\right),$$

where $\tau_{true}$ is the true shear stress (Pa) after the correction, M is the measured torque (N·m), R is the radius of the measuring system (m), and ${\dot{\gamma }}_{R}$ is the shear rate at the rim of the geometry (s^−1^).

**Figure 2:**
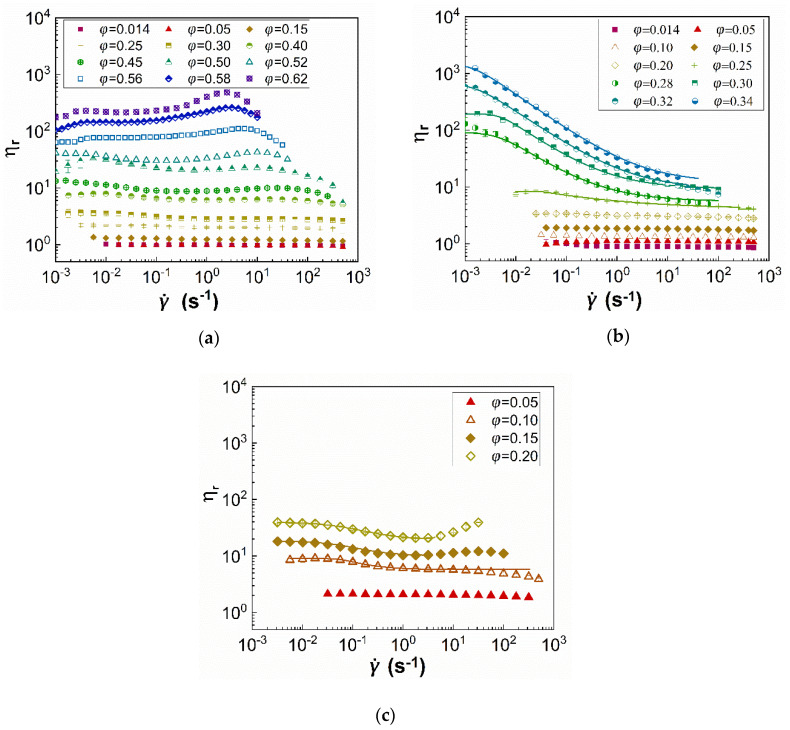
Relative viscosity ($\eta_{r}$) of (**a**) GS2; (**b**) PS52; and (**c**) PS226 suspensions in glycerol as a function of shear rate ($\dot{\gamma }$) and particle volume fraction (φ). Continuous lines correspond to the Carreau fittings to the data.

The density of the silica particles is different from that of glycerol, as shown in Table 1, and thus shear stresses above a critical level need be applied during the rheological measurements to exclude any particle sedimentation effects. This stress value can be estimated using the dimensionless Shields parameter [28]:

(2) $$Sh=\frac{\tau }{2a\Delta \rho g},$$

where τ is the shear stress (Pa), a the particle radius (m), Δρ the difference in densities between the particles and the fluid (kg/m^3^), and g the gravitational acceleration (m/s^2^). Sh in Equation (2) is effectively the ratio of the fluid induced force acting on the particle to the particle weight; a cut off value of Sh=1 is assumed to estimate the critical stress beyond which particle sedimentation can be neglected. The values of this critical stress are 0.01 and 0.09 Pa for the GS2 and the PS52/PS226 particles, respectively; thus, the viscosity values at shear stresses below these values are excluded from the flow curves in Figure 2. 

The estimated *Pèclet* numbers ($Pe=6\pi \alpha^{3}\dot{\gamma }\eta_{f}/k_{B}T$, where α is the average particle radius) based on the average particle diameter vary from 10^3^ to 10^9^, i.e., they are sufficiently large to exclude Brownian motion. However, due to the high polydispersity of the particles used here, their size can extend into the submicron scale, i.e., d<1 μm. The Brownian effects on suspension rheology were evaluated by estimating the critical shear rate at which less than 1% of the particle volume are able to induce Brownian motion assuming Pè=10 [29]; the estimated shear rates were $\dot{\gamma }=0.04\;s^{-1}$ for the GS2, $\dot{\gamma }=0.004\;s^{-1}$ for the PS52 particles, while for the PS226 particle suspensions the corresponding shear rate was below $\dot{\gamma }=0.001\;s^{-1}$. Therefore, Brownian motion effects are likely to occur only at specific cases of highly concentrated suspensions and at very low shear rates. In the case that shear rates as low as $\dot{\gamma }=0.001\;s^{-1}$ can be used without any sedimentation effects taking place, the fraction of particles able to induce Brownian motion is found equal to 9.5% for the GS2 and 5.7% for the PS52. The estimated value for the PS226 suspensions is negligible, since only 0.3% of the particles in the suspensions are in the colloidal regime at $\dot{\gamma }=0.001\;s^{-1}$. 

The Carreau model captured quite well the shear thinning response of the concentrated suspensions of PS52 and PS226 suspensions in glycerol and its equation is given below [30]:

(3) $$\eta =\eta_{\infty }+\left(\eta_{0}-\eta_{\infty }\right){\left(1+{\left(\lambda \dot{\gamma }\right)}^{2}\right)}^{\frac{n-1}{2}},$$

where $\eta_{0}$ and $\eta_{\infty }$ are the zero- and infinite-shear rate viscosities (Pa·s), respectively; λ is the relaxation time (s); and n is the flow index, indicative of the shear thinning degree [31]. 

The Quemada model was employed to describe the relation between the relative viscosity of the suspensions and the particle volume fraction [32]:

(4) $$\eta_{r}={\left(1-\frac{\varphi }{\varphi_{m}}\right)}^{-2},$$

where $\varphi_{m}$ is the maximum packing fraction.

## 3. Results

### 3.1. Steady State Shear Rheology

The relative viscosity ($\eta_{r}$) values of the three types of suspensions i.e., GS2, PS52, and PS226 in glycerol is illustrated in Figure 2 as a function of shear rate ($\dot{\gamma }$) for various particle volume fractions (φ). The results illustrate a non-monotonic influence of the particle specific surface area on the behaviour of the suspensions. The PS52 particles are characterized by a pronounced shear thinning behaviour as the volume fraction increases (Figure 2b), with the onset observed at φ≥0.25, whereas glass spheres and PS226 particle suspensions exhibit almost negligible or weak shear thinning (Figure 2a,c) at φ≥0.30 and φ≥0.10, respectively. The shear thinning response of the GS2 and PS226 is also followed by shear thickening at higher particle volume fractions (φ≥0.50 and φ≥0.15), while no evidence of shear thickening is observed for the PS52 suspensions, even at the highest particle volume fractions studied. The PS226 suspensions (Figure 2c) also exhibit the highest relative viscosities among the three particle suspensions. The shear thinning response of the silicas can be described by the Carreau equation (Equation (3)) sufficiently well and the corresponding fittings are shown as continuous lines in Figure 2. 

**Figure 3:**
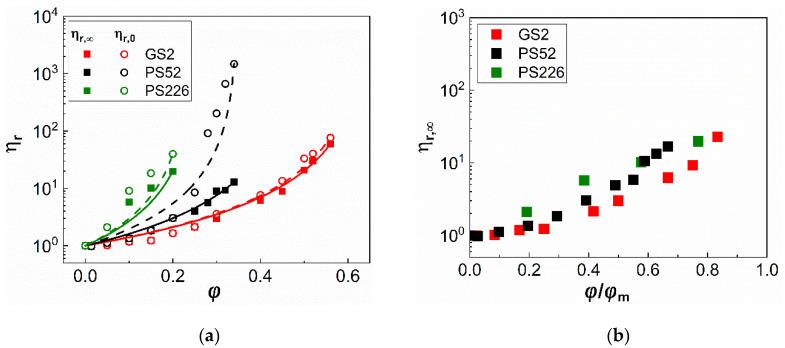
(**a**) Zero shear rate ($\eta_{r,0}$) and infinite shear rate ($\eta_{r,\infty }$) relative viscosities of the three types of particle suspensions as a function of particle volume fraction (φ). The continuous and dashed lines correspond to the Quemada fittings to the experimental data (Equation (4)); (**b**) Infinite shear rate ($\eta_{r,\infty }$) relative viscosity values of all suspensions as a function of the particle volume fraction (φ) normalised with $\varphi_{m,\infty }$.

The Carreau fittings are used to estimate the zero- and infinite-shear rate relative viscosities, $\eta_{r,0}$ and $\eta_{r,\infty }$, respectively. Their values are summarised in Figure 3a as a function of φ; the Quemada (Equation (4)) fittings are also included indicated by continuous and dashed lines. The $\eta_{r,0}$ values for the glass sphere suspensions were determined at $\dot{\gamma }=10^{-3}s^{-1}$. Similarly, the $\eta_{r,\infty }$ values for suspensions exhibiting both shear thinning and shear thickening, were taken in the shear rate region, just before the onset of the shear thickening regime. It can be seen that the relative viscosity values at zero and infinite shear ($\eta_{r,0}$ and $\eta_{r,\infty }$) almost overlap for the glass sphere suspensions, since they exhibit negligible shear thinning. In contrast, they differ substantially for the PS52 particle suspensions illustrating their pronounced shear thinning at φ≥0.25 and only slightly for the PS226 ones due to their mild shear thinning behaviour. The PS52 and PS226 particles both increase the viscosity of glycerol to a greater extent than glass spheres due to their surface asperities, which offer a higher area available for particle contacts [3,9], and porosity with the PS226 inducing a much steeper increase at lower particle volume fractions as compared with the other two particle types. 

Table 2 summarises the maximum packing fraction ($\varphi_{m}$) obtained from the Quemada fittings to the relative viscosities in the infinite shear rate regimes $\eta_{r,\infty }$. Increasing the specific surface area of the particles results in a decrease in the $\varphi_{m}$ values. The glass sphere (GS2) suspensions exhibit the highest values of $\varphi_{m}$ out of the three suspensions and these are similar to those reported in the literature for randomly closed packed monodisperse and polydisperse spheres [33,34,35]. The $\varphi_{m}$ values for both the PS52 and PS226 particles are lower as compared with the GS2. This can be attributed to the increased particle contacts enabled by their irregular surfaces which can limit the particle loading capacity. The origins of particle contacts and their relevance to the observed suspension rheology are discussed in more detail in Section 4.

Figure 3b replots the $\eta_{r,\infty }$ values as a function of the particle volume fraction, this time normalised with the maximum packing fraction ($\varphi_{m}$) at infinite shear rate. Although normalising φ nearly collapses the relative viscosities of the three types of suspensions, small variations in the $\eta_{r,\infty }$ values between the glass spheres (GS2) and the irregular commercial silicas (PS52 and PS226) can still be discerned, highlighting the strong effect of particle surface characteristics on suspension rheology. 

In our previous work [12], we showed that the strong shear thinning behaviour of the PS52 suspensions, as opposed to that of GS2, can be attributed to the surface asperities being elastically deformed due to the frictional contacts between the former particles. According to a theory introduced by Chatté et al. [36] and Lobry et al. [28], this leads to a decrease in the microscopic friction coefficient and shear thinning. The shear thinning of the glass spheres is, however, suppressed due to the presence of a solvation layer as the hydroxyl groups (-OH) of the glycerol molecules bind onto the inherently hydrophilic silicas through hydrogen bonding. The PS226 particles are also irregularly shaped, similar to the PS52, as shown in Figure 1, and exhibit much higher specific surface area as compared with the latter; thus, one would expect strong shear thinning behaviour for these particles at even lower particle concentrations as compared with PS52 ones. However, it seems that the increased specific surface area of the PS226 competes with the frictional contacts induced by the particle irregular surface and leads to the PS226 suspensions exhibiting weak shear thinning behaviour, followed by shear thickening at the highest φ values. This might be associated with the limited loading capacity of the PS226 particles as compared with the other two particle types, as only volume fractions up to φ=0.20 could be achieved. It should be noted that the solvation layer, which acts as a lubricant to particle contacts, exists for all types of particle suspensions, but its effectiveness appears to depend on the particle surface area as indicated by the non-monotonic results reported herein. 

**Figure 4:**
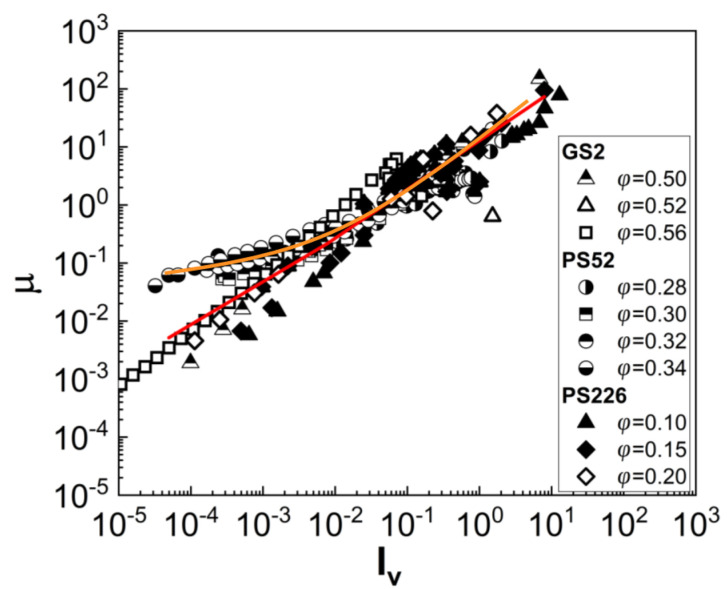
Macroscopic friction coefficient (μ) of selected suspensions as a function of the viscous number. The continuous lines are guides to the eye for the two regimes of friction coefficient values.

To further probe the frictional contacts between the different types of particles, the friction coefficients (μ) were estimated using the bulk rheology data. The friction coefficient was estimated by the ratio of the shear stress (τ) to the particle pressure (P), assumed equal to the normal stress as measured with the rheometer [9,37,38]. Figure 4 plots the estimated μ values for selected and concentrated suspensions of the three particle types as a function of a normalised shear rate, namely the viscous number ($I_{v}=\eta_{f}\dot{\gamma }/P$, where $\eta_{f}$ is the viscosity of the suspending medium, glycerol, $\eta_{f}=1.3\;Pa\cdot s$). Two distinct regimes can be observed in the friction coefficient (μ) values in the low $I_{v}$ region, which seem to be related to the extent of shear thinning in each case; while the coefficient estimates of the three types of particles seem to match and vary linearly with $I_{v}$ for $I_{v}\geq 0.01$, a deviation is observed for lower viscous numbers. An asymptotic behaviour characterized by higher μ values is exhibited by the PS52 suspensions reaching a value of μ≈0.02 as $I_{v}$ tends to 0; this behaviour indicates the presence of frictional contacts and explains the strong shear thinning response of these suspensions, despite the relatively low particle volume fraction as compared with the GS2 suspensions. The asymptotic value estimated for PS52 is lower than that reported by Boyer et al. (2011), i.e., μ=0.32, for PMMA (polymethylmethacrylate, d=1.1±0.05 mm) and PS (polystyrene, d=0.58±0.01 mm) spheres suspended in index-matched Newtonian fluids at room temperature. This may be attributed to the smaller size of the particles used in the present study (d=17.5±15.5 μm) and the assumptions of the particle pressure being equal to the measured normal stress; local particle pressure can also be influenced by particle surface asperities and porosity. It should be recalled that, in the study of Boyer et al., the particle pressure was directly measured by a pressure-imposed shear cell [37]. 

On the contrary, no asymptotic μ value was observed for the GS2 and PS226 suspensions, which is consistent with their low, almost negligible degree of shear thinning. However, as PS226 particles exhibit similar surface morphology to the PS52 particles (Figure 1a,b), some frictional contacts are expected. The absence of such contacts inducing a shear thinning response indicates a more complex rheology for the highly porous PS226 particles and highlights the need for more parameters to be explored to fully explain it; these are discussed in Section 4. It can, thus, be concluded from Figure 4 that although the PS52 suspensions are in the frictional regime, a lubrication effect seems to govern the rheology of the PS226 suspensions similar to that of the highly concentrated glass sphere suspensions. The deviations of μ values from a single master curve, as shown in Figure 4, have generally been observed in numerical studies with varying dynamic friction coefficients, highlighting the strong effects of friction on suspension rheology [38]. 

Porous particles further complicate suspension rheology as part of the solvent becoming absorbed into the pores and leading to an increase in the effective particle volume fraction as compared with the apparent one. The effective volume fraction of the suspensions, $\varphi_{eff}$, is given by [15]:

(5) $$\varphi_{eff}=\frac{w_{p}}{\frac{\rho_{p}}{\rho_{susp}}-\varepsilon w_{p}\frac{\rho_{p}}{\rho_{f}}}$$

where, $w_{p}$ is the weight fraction of the particles in suspension, ε represents the particle porosity, $\rho_{p}$ is the density of the solid particles, $\rho_{f}$ the density of the suspending medium, and $\rho_{susp}$ is the suspension density defined as [39]:

(6) $$\rho_{susp}=\varphi \left(\rho_{p}-\rho_{f}\right)+\rho_{f}$$

with φ the apparent particle volume fraction. 

**Figure 5:**
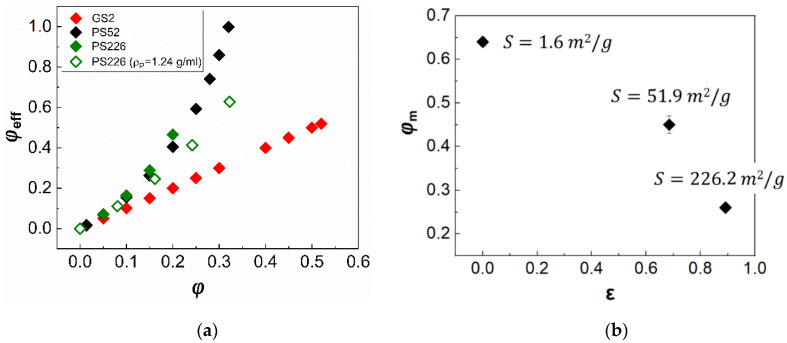
(**a**) Effective volume fraction ($\varphi_{eff}$) plotted as a function of apparent particle volume fraction (φ) and (**b**) maximum packing fraction of all suspensions as a function of porosity (ε); the specific surface area (S) values are also shown in the figure. The $\varphi_{eff}$ of the PS226 particles was estimated using both the material density ($\rho_{p}=2\;g/mL$) (green filled diamonds) and the experimentally measured density ($\rho_{p}=1.24\;g/mL$) (green open diamonds).

The estimated $\varphi_{eff}$ values are presented in Figure 5a as a function of the apparent volume fraction, alongside a plot of the maximum packing fraction, $\varphi_{m}$, as a function of particle porosity, ε, and specific surface area, S (Figure 5b). Two sets of data are shown in Figure 5a for the PS226 particles. These refer to the $\varphi_{eff}$ estimates using either the material’s density (filled green diamonds), i.e., $\rho_{p}=2\;g/mL$, or the experimentally derived particle density, i.e., $\rho_{p}=1.24\;g/mL$, through volumetric measurements of the weighted particle. A volumetric cylinder was filled with a known volume of water, and then 1 gr of weighted mass was added each time and the increase in the water volume was recorded. The experimentally derived density of the PS226 particles is considerably lower as compared with the pure silicon dioxide density probably due to the high porosity of these particles. Interestingly, the $\varphi_{eff}$ values for the PS226 using $\rho_{p}=1.24\;g/mL$ appear lower as compared with that of the PS52 independently of the higher porosity of the former. This is because not only the density values in Equations (5) and (6) but also the apparent volume fraction needs be recalculated to correspond to $\rho_{p}=1.24\;g/mL$. Therefore, the increased $\varphi_{eff}$ of the PS52 and PS226 suspensions as compared with the glass spheres due to particle porosity, can be responsible for the onset of non-Newtonian rheological phenomena at lower apparent φ values for the commercial silicas, as shown in Figure 2. The $\varphi_{m}$ values also appear to decrease with particle porosity and specific surface area as expected due to an increase in the available particle-particle and particle-solvent areas for contact. 

The extent of shear thinning and shear thickening response of the suspensions exhibiting non-Newtonian rheology, as shown in Figure 2, is further described through the parameters $\eta_{r,e}$ (Equation (7)) and $\eta_{r,t}$ (Equation (8)), respectively. The $\eta_{r,e}$ is defined as:

(7) $$\eta_{r,e}=\frac{\eta_{r,0}-\eta_{r,\infty }}{\eta_{r,\infty }},$$

$\eta_{r,t}$ is defined in a similar manner:

(8) $$\eta_{r,t}=\frac{\eta_{r,peak}-\eta_{r,\infty }}{\eta_{r,\infty }},$$

with $\eta_{r,peak}$ representing the peak relative viscosity in the shear thickening area. 

**Figure 6:**
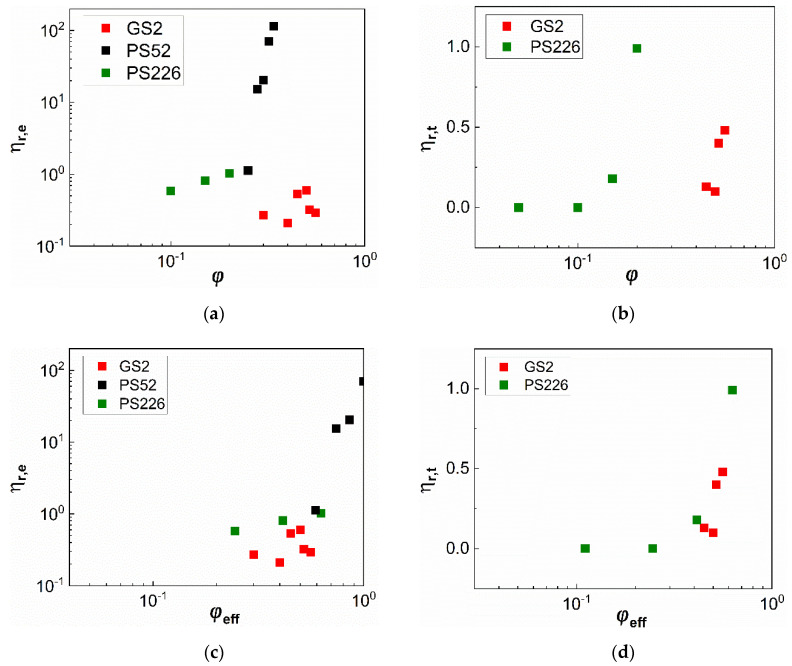
Extent of (**a**) shear thinning ($\eta_{r,e}$) and (**b**) shear thickening ($\eta_{r,t}$) of the suspensions exhibiting non-Newtonian rheology as a function of particle volume fractions (φ); (**c**,**d**) represent the same data with (**a**,**b**) as a function of the effective particle volume fraction ($\varphi_{eff}$). In the case of PS226, the $\varphi_{eff}$ values were estimated using the experimentally derived density.

The dependence of these parameters on the particle volume fraction is presented in Figure 6a,b. respectively. The GS2 suspensions exhibit almost negligible shear thinning, while the PS52 particle suspensions exhibit the most pronounced shear thinning as compared with the other two types of particles. Shear thickening (Figure 6b) is only observed for the GS2 and PS226 suspensions, with the latter showing higher $\eta_{r,t}$ values. 

As discussed above, particle porosity influences interparticle interactions, and thus suspension rheology through increasing the effective volume fraction of the particles in the suspension. By replotting the $\eta_{r,e}$ and $\eta_{r,t}$ values as a function of $\varphi_{eff}$ for the two silicas, they appear to approach those of the glass sphere suspensions (Figure 6c,d, respectively). However, considering only porosity as the main factor to estimate the effective volume fraction of the suspensions is not adequate to fully capture their non-Newtonian behaviour. The irregular silicas still show stronger shear thinning and shear thickening response as compared with the non-porous glass spheres despite scaling the data by $\varphi_{eff}$. 

### 3.2. Viscoelasticity

**Figure 7:**
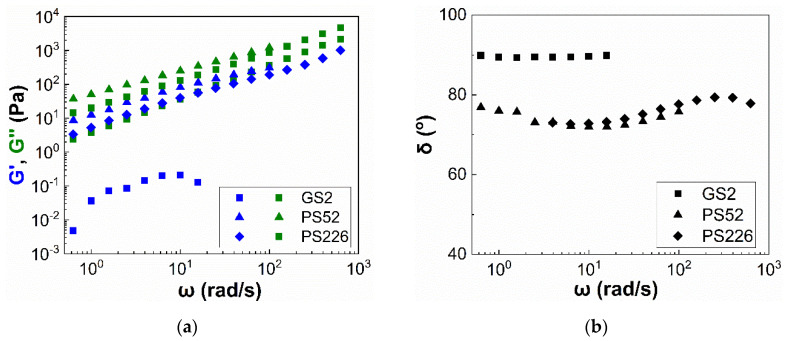
(**a**) Viscoelastic moduli ($G^{\prime }$, $G^{\prime \prime }$) and (**b**) phase angles (δ) of the three types of particle suspensions in glycerol as a function of the angular frequency (ω) at a fixed particle volume fraction of φ=0.30. The suspension at φ=0.20 is used for the PS226 particles, as this is the highest particle volume fraction achieved to provide a homogeneous sample by visual inspection.

Particle suspensions are likely to exhibit viscoelastic properties at sufficiently high particle volume fractions, in which interparticle interactions are strong giving rise to the elastic component of the material. The viscoelastic properties of selected highly concentrated suspensions of the three particle types were investigated under oscillatory frequency sweeps in the linear viscoelastic region (strain amplitude, $\gamma_{0}=0.1\%$). The measured viscoelastic moduli, i.e., storage, $G^{\prime }$, and loss modulus, $G^{\prime \prime }$, are presented in Figure 7a as a function of the angular frequency (ω) for all three suspensions at a fixed particle volume fraction of φ=0.30. The suspension at φ=0.20 is used for the PS226 particles, as this is the highest volume fraction achieved to provide a homogeneous sample based on visual inspection. The suspensions exhibit viscous dominated behaviour, with the $G^{\prime \prime }$ values being higher than the $G^{\prime }$, at all experimental conditions and particle characteristics, indicating the hindrance of direct particle contacts due to the presence of the solvation layer [23] in the whole range of angular frequencies investigated. Increasing the particle specific surface area leads to increasing both $G^{\prime }$ and $G^{\prime \prime }$ values as compared with the glass sphere suspension. However, the PS226 exhibit slightly lower values of the viscoelastic moduli as compared with the PS52, which can be attributed to the lower $\varphi_{eff}$ of the former.

The suspension viscoelasticity can be further evaluated from the corresponding phase angles (δ) obtained through the frequency sweeps (Figure 7b). In general, a value of δ≈0° indicates that the material behaves as an elastic solid, while δ≈90° represents a liquid-like behaviour. The phase angle values, varying from 0° to 90°, indicate that the material shows properties between a solid and a liquid, i.e., it is viscoelastic. The GS2 suspension shows δ values very close to 90° and which are almost independent of the applied angular frequency. In contrast, PS52 and PS226 exhibit lower phase angles (δ≤80°), indicating an increase in suspension viscoelasticity. The lower phase angles for the PS52 can originate from interparticle interactions also responsible for the shear thinning response of these suspensions at low shear rates under steady state (Figure 2b). The low phase angles observed for the PS226 suspension, similar to those of the PS52, might also arise from the weak shear thinning of the former. 

## 4. Discussion

Commercial precipitated silicas are highly irregular (rough) and porous, thus, far from idealized particles commonly studied in suspension rheology. In an attempt to explain the effects of their surface characteristics on the measured suspension rheology, the particle specific surface area (S) is utilized. A non-monotonic effect of S on the rheology of the suspensions under investigation is observed arising from the competing mechanisms of particle–particle and particle–solvent interactions; the latter is associated with the existence of a solvation layer around the silicas by the glycerol molecules. 

Although we were not able to measure the thickness of the solvation layer in our study, we postulate that it is higher than the 3 nm measured by Gao et al. [23] for an aqueous solution. This is due to glycerol having three hydroxyl groups (-OH) on its molecule to bind on the silica surface in contrast to water which has one. The presence of the solvation layer appears to hinder the shear thinning response of the glass sphere suspensions at 20 °C, while shear thickening is only observed at sufficiently high particle volume fractions close to jamming. This behaviour is in contrast to observations of other researchers using non-Brownian glass sphere suspensions in aqueous polar media, such as Saint-Michel et al. [20] and Amiri et al. [19]. The viscous dominated response in the oscillatory frequency sweeps is also indicative of the absence of direct interparticle contacts or particle agglomeration [7]. 

Unlike the glass sphere suspensions, the PS52 particles exhibit the onset of shear thinning response at much lower particle volume fractions (φ≥0.25), attributed to a frictional shear thinning mechanism [12], based on the elastic deformation of the surface asperities during particle contact, resulting in a decrease in the microscopic friction coefficient with shear rate [9,28,36]. Thus, particle surface asperities seem to compete with the interparticle interaction screening induced by the glycerol layer, and in the case of PS52 particles, the surface asperities seem able to penetrate the solvation layer inducing particle contacts. 

Although the PS226 suspensions exhibit the onset of shear thinning response at φ≥0.10, its degree is much less pronounced as compared with PS52, while shear thickening also occurs at the higher particle volume fractions (φ=0.15 and 0.20). It should be recalled that only concentrations up to φ=0.20 could be achieved for this type of suspensions to obtain a homogeneous sample. The lower effective volume fraction of PS226, estimated at $\rho_{p}=1.24\;g/mL$ as compared with the PS52 particles can be one reason for this behaviour. In addition, the PS226 suspensions do not show a critical stress which is independent of φ for the onset of the shear thinning response, whose values are shown in Appendix A, in contrast to the PS52 suspensions [12], and thus the friction driven mechanism is inadequate to fully explain their weak shear thinning. Shear thickening is also observed at φ≥0.15, exhibiting a stronger extent (higher $\eta_{r,t}$ values, Figure 6d) as compared with that of the glass spheres, even after scaling the data with $\varphi_{eff}$. This may be attributed to the much higher specific surface area of PS226 particles as compared with the GS2 linked with a higher density of free surface silanol groups ($\delta_{OH}$) and inducing more pronounced jamming effects and shear thickening. 

The density of the free surface silanol groups ($\delta_{OH}$) can significantly influence interparticle interactions, and thus suspension rheology and shows a linear increase with S [16,18] as follows:

(9) $$\delta_{OH}=\frac{\alpha_{OH}S}{N_{A}},$$

where $\delta_{OH}$ is the density of the surface silanol groups (mol OH/g of SiO_2_), $a_{OH}$ is a constant equal to 4.6 OH/nm^2^, S is the particle specific surface area (m^2^/g), and $N_{A}$ is the Avogadro constant equal to 6.023 × 10^23^ atoms/mol. The estimated densities of the silanol groups for the three types of particles are presented in Table 3. As expected, $\delta_{OH}$ values increase as the specific surface area increases from $S=1.6\;m^{2}/g$ for GS2 to $S=51.9\;m^{2}/g$ for PS52 and $S=226.2\;m^{2}/g$ for PS226. 

The impact of increasing the silanol groups on the surface of the silicas can be two-fold. On the one hand, it can increase the strength of the solvation layer formed by the glycerol molecules due to providing a higher number of available contact points between the particles and the suspending medium [21]. On the other hand, a higher density of silanol groups, due to increased specific surface area results, can promote interparticle interactions leading to increased suspension viscosity and non-Newtonian rheological behaviour. Suspension rheology results from the competition between these two phenomena based on the particle-particle or particle-solvent affinity; these two phenomena often act synergistically and their individual effect on suspension rheology is hard to be distinguished.

For example, the high density of the surface silanol groups on the PS226 particles might enhance the strength of the solvation layer promoting short range repulsive forces between the particles and inducing shear thickening at sufficiently high shear rates [21]; this can also prevent the frictional contacts at low shear, and thus explain the suppressed shear thinning response. The latter might stem from the transient deformation of the solvation layer with shear as the particles weakly sense the presence of neighboring particles. The viscoelastic response of the PS226, similar to PS52 suspension, can also be explained through the formation of a stable network between the PS226 particles and glycerol. In contrast, in the case of the PS52 suspensions, the $\delta_{OH}$ value might not be adequate to induce such phenomena. 

## 5. Conclusions

This study showed a non-monotonic effect of the particle specific surface area (S) on the rheology of non-Brownian silica particle suspensions. S was utilized to express the combined effects of particle surface asperities and porosity. Three types of silica particles with varying surface morphology and porosity were suspended in glycerol and the rheological properties of their suspensions were investigated under steady state and oscillatory shear measurements. The presence of a solvation layer formed by the highly viscous glycerol hindered the shear thinning response of the GS2 suspensions under steady state shear, while, in contrast, pronounced shear thinning was observed for the PS52 suspensions. It was postulated that the surface asperities on the PS52 surface are able to penetrate this solvation layer inducing frictional contacts between the particles and leading to pronounced shear thinning. The frictional contacts between the PS52 particles were also demonstrated by the estimation of the microscopic friction coefficients using the measured shear and normal stresses. 

Despite their much higher specific surface area, the PS226 suspensions, exhibited similar trends to the glass sphere suspensions, i.e., shear thinning followed by shear thickening, but at remarkably lower particle volume fractions as compared with the GS2 particles. The observed rheological phenomena were likely to arise from a competition between the effective volume fraction due to particle porosity and the increased density of the surface silanol groups of the PS226 particles; the latter is critical parameters to controlling the (repulsive) interparticle hydration force induced by glycerol. 

This study demonstrates the role of the specific surface area in tuning the rheology of non-Brownian silica particle suspensions, a versatile particle system employed in a range of industrial formulations. Further studies with well controlled particle surface characteristics (surface asperities and porosity) will aid a deeper understanding of the behaviour of such complex systems and the generalization of our findings. 

## Figures and Tables

**Table 1:** Particle and solvent characteristics.

Particle Properties	GS2	PS52	PS226
Density ($\rho_{p},\;g/mL$)	1.1	2.0	2.0
Diameter (*D*, μm)	11 ± 5.5	17.5 ± 15.2	12.5 ± 4.0
Specific surface area (*S*, m^2^/g) *^1^	1.6	51.9	226.2
Porosity *^1^ (ε)	-	68.5%	89.2%
**Fluid properties at 20 °C**	**Glycerol**		
Density ($\rho_{f},\;g/mL$)	1.25		
Viscosity ($\eta_{f},\;Pa\cdot s$)	1.3 ($\eta_{f,G}$ *^2^)		

*^1^ Estimated from the BET method and *^2^ viscosity of glycerol.

**Table 2:** Maximum packing fraction ($\varphi_{m}$) of all suspensions in glycerol as obtained from the Quemada fittings to the infinite shear rate relative viscosities, $\eta_{r,\infty }$.

Particles	$\varphi_{m}$
GS2	0.64
PS52	0.47
PS226	0.26

**Table 3:** Density of the surface silanol groups of all the three types of particles used in the present study.

Particles	$\delta_{OH}$ $\left(mol\;OH/g\;of\;SiO_{2}\right)$
GS2	0.012
PS52	0.40
PS226	1.70

## Appendix A

Table A1 shows the relaxation time (λ) estimated from the Carreau fittings to the viscosity data of the filler silicas in Figure 2c, alongside the viscosity values (η) and the corresponding shear rates ($\dot{\gamma }$) used to estimate the critical stress ($\tau_{c}$) for the onset of shear thinning according to the theory presented in our recent work for the abrasive silica suspensions [12]. It can be seen that the $\tau_{c}$ values are highly dependent on the particle volume fraction highlighting the more complex rheology induced by the PS226, which cannot be described by a single $\tau_{c}$.

**Table A1:** Relaxation time (λ) as derived from the Carreau fittings in Figure 2c. Viscosity values (η) at the specified shear rates ($\dot{\gamma }$) for the estimation of the critical stress values ($\tau_{c}$) of the filler silica suspensions investigated in the present study.

φ	0.10	0.15	0.20
λ (s)	14.99	12.98	8.9
η	6.27	10.64	22.84
$\dot{\gamma }\;\left(s^{-1}\right)$	0.56	0.56	0.56
$\tau_{c}$ (Pa)	0.54	1.07	3.34
